# How do European Mature Adults and Elderly Perceive SARS-COV-2 and Associated Control Measures? A Cross-Country Analysis of Mental Health Symptoms in June and July 2020

**DOI:** 10.3389/ijph.2022.1604218

**Published:** 2022-02-23

**Authors:** Julian Perelman, Miguel Xavier, Pedro Pita Barros

**Affiliations:** ^1^ Center for Research in Public Health, National School of Public Health, New University of Lisbon, Lisbon, Portugal; ^2^ Comprehensive Health Research Centre, Universidade NOVA de Lisboa, Lisbon, Portugal; ^3^ NOVA Medical School, Faculty of Medical Sciences of Lisbon, New University of Lisbon, Lisbon, Portugal; ^4^ National Mental Health Program, General-Directorate for Health, Lisbon, Portugal; ^5^ School of Business and Economics, New University of Lisbon, Lisbon, Portugal

**Keywords:** mental health, COVID-19, Europe, mature adults, control measures

## Abstract

**Objectives:** Recent literature points out that elderly people are psychologically resilient to COVID-19, but the studies were performed in specific contexts. We measured the link between the worsening of mental health symptoms, the epidemiologic situation, and control measures among European people aged 50 or older.

**Methods:** We used data from the 2020 wave of SHARE, merged with Oxford COVID-19 Government Response Tracker data (*n* = 38,358). We modeled the risk of worsening of depression, anxiety, sleeping trouble, and loneliness symptoms’ self-perception, as functions of control measures and 7-days death incidence, using logistic regressions.

**Results:** The worsening of anxiety and depression perception were more common (16.2 and 23.1%, respectively), compared to that of sleeping troubles and loneliness (8.1 and 11.5%, respectively). The worsening of depression and anxiety perception was negatively related to the rigor of control measures. The seven-days death incidence was positively linked to all symptoms except sleeping troubles.

**Conclusion:** Older people were the most exposed to death risk and were affected psychologically by the COVID-19 epidemiological situation; yet control measures were protective (or neutral) to their mental health condition.

## Introduction

The recent literature has pointed out that elderly people were resilient to SARS-COV-2 (COVID-19) and to the control measures implemented to combat the pandemic. Arguments were that elderly people would better cope with isolation, and that this was related to the ability to maintain meaningful relationships and to a greater “wisdom” comprising emotional regulation, empathy and acceptance of uncertainty [1].

A longitudinal study in the UK showed that in comparison to an 18.9-percent prevalence 2018–2019, 27.3% reported mental distress in April 2020; but compared to earlier trends, the deterioration in mental health was significant only among those younger than 35. A study on three cohorts in the Netherlands compared mental health disorders in April and May 2020 to those observed in previous waves (between 2006 and 2016) [2]. Among those without previous mental health disorders, a modest increase in depression, anxiety, and loneliness scores was observed, while no change was measured among those with previously diagnosed disorders. A longitudinal study on Dutch people older than 62 also observed that, despite the increase in emotional loneliness, depression and anxiety did not worsen in May 2020 compared to October–November 2019 [3]. The authors attributed the absence of deterioration to the low death incidence, the adequate hospital response, and the weak restriction model adopted in Netherlands.

Although not assessing changes by following people across time, cross-sectional studies highlighted similar patterns. A web-based survey carried out in June 2020 in the United States observed a 24.3-percent prevalence of depressive disorder and a 25.5-percent prevalence of anxiety disorder, compared to 6.5% and 8.1%, respectively, observed in another cross-section study in 2019 [4]. The prevalence of depression or anxiety disorder was, however, five times lower among those aged 65 or above compared to those aged 25–44. A cross-section web-based study in Spain during the first months of the pandemic measured an 18.7-percent prevalence of depression and a 21.6-percent prevalence of anxiety that was significantly lower among those aged 60–80, compared to younger people [5]. A similar study in Spain in March and April 2020 confirmed the lower mental distress among people older than 60 [6].

Nevertheless, these studies were performed in specific contexts, marked not only by different rates of infection and deaths, but also by heterogeneity in the control measures. In particular, studies were limited in their capacity to estimate the discrepancies of the mental health burden across sociodemographic categories, or to relate this burden to the country-specific situation, in regard to the severity of the pandemic or that of public health policies.

This study uses the last wave of the SHARE project (SHARE-COVID19), which was applied during the year 2020 to a representative sample of European mature adults, to measure the link between the worsening of mental health symptoms’ self-perception and the epidemiologic situation and control measures.

## Methods

### Data

We used data from the Survey on Health, Aging, and Retirement in Europe (SHARE), which followed people older than 50 from 2004 to 2020 (8 waves, the last in 2020). Individuals were randomly selected from national or regional population registries. In the latter case, two‐ or multi‐stage designs were used, in which regions were sampled first and then individuals randomly selected within regions (for more information on the survey design, see http://www.share-project.org/data-documentation/methodology-volumes.html). We used data from the 2020 wave, which took place between March and August 2020, that is, after the first cases of COVID-19 were diagnosed in Europe. The following 27 countries were included: Belgium, Bulgaria, Croatia, Cyprus, Czech Republic, Denmark, Estonia, Finland, France, Germany, Greece, Hungary, Israel, Italy, Latvia, Lithuania, Luxemburg, Malta, Netherlands, Poland, Portugal, Romania, Slovakia, Slovenia, Spain, Sweden, and Switzerland.

The original sample was formed by 53,499 individuals surveyed during wave 8, among whom 45,563 were interviewed with the COVID-specific questionnaire in June and July 2020. We restricted our sample to people over 50, for whom the SHARE survey ensured representativeness (*n* = 45,384).

We then merged this sample with data from Oxford COVID-19 Government Response Tracker, which includes information on containment and closure policies, economic policies, and health system policies [7]. The merging was performed by attributing the COVID-19-related variables to each individual according to her country and interview date. In other words, each individual was characterized by the epidemiological situation and control measures in her country at the moment she was interviewed. Information was missing for Spain, Switzerland, Israel, Latvia, and Malta, i.e., 6,205 observations were missing, so that a more restricted sample of 38,358 observations was analyzed.

### Outcomes

We created binary variables for the worsening perception of sadness, sleeping difficulties, and feelings of depression, anxiety, nervousness, and loneliness. To do so, we coded as “1” those who declared having had these troubles in the last month and declared that the troubles have worsened due to COVID-19. The comparator (“zero” value) was thus those who either declared an absence of trouble, or its presence but without worsening due to COVID-19.

### Explanatory Variables and Covariates

Our main explanatory variable was the stringency index, which characterized the rigor of control measures using a score based on eight items: school closing, workplace closing, canceling of public events, restrictions on gatherings, closed public transport, stay-at-home requirements, restrictions on internal movements, and international travel controls. Each item includes from three to five categories, from the least to the most severe restriction. The index is constructed as the sum of the scores, reordered on a 0–100 scale, with additional scores if the policy had been implemented nationwide (vs regional or local implementation) [7]. We also considered the incidence of COVID-19 deaths in the 7 days prior to the interview, per million habitants, available from the Oxford COVID-19 Government Response Tracker, which retrieved these data from the European Center for Disease Control (ECDC).

We included as covariates age and sex categories, the living condition (alone or not), the working condition before COVID-19 (worker or non-worker), and the occurrence of an adverse health event since 2017 (diabetes, hip fracture, cancer, hypertension, chronic lung disease, heart disease). The presence of chronic events was considered as a potential confounding factor because mental health is likely to be affected by the occurrence of such diseases. A systematic review showed, for example, that the risk of depression is 45% higher for each additional chronic condition, and that the risk is twice higher when experiencing multimorbility [8]. We could not adjust for education because of the small heterogeneity among our sample, and its close association with age. We also excluded the self-reported health variable, which is known to be related with depression symptoms and could therefore be redundant [9].

### Statistical Analysis

All dependent variables were modeled using logistic regressions (with robust standard errors) and reported as marginal effects. The marginal effects (risk or rate differences for epidemiologists) is the difference between the observed risks (proportions of individuals with the outcome of interest) across different groups. The marginal effect is interpreted as the estimated absolute difference in the probability of experiencing the event. As marginal effects vary across individuals (their baseline risk and other characteristics), they were calculated from logistic regressions, for a given variable, assuming the average value for all other covariates (for more information, see [10]).

Stringency and death incidence variables were interacted with age and sex categories, living and working condition, and close death, in order to check whether COVID-19 and public health measures had different effects on different subpopulations.

All analyses included country fixed effects. On the one hand, these variables may capture part of the stringency and death effects, because these were measured at the country level and were relatively stable during the period of observation. On the other hand, the literature has long shown the considerable heterogeneity of mental health across Europe related to treatment access and patterns, socioeconomic context, and cultural dimensions. Not including country fixed effects as covariates may lead us to overestimate the stringency and death effects, which may capture part of these unobserved country characteristics, and we therefore opted for a more conservative approach.

All analyses were conducted using Stata version 13.1 (StataCorp, College Station, TX, United States).

## Results

Most of the people were aged between 65 and 79 years old (52.8%), were women (58.6%), non-workers (79%), and not living alone (75.1%) (Table 1). A small minority had recently experienced a COVID-19-related death or hospitalization of a person close to them (2.3 and 3.1%, respectively). The emergence of morbidities in the last 3 years affected less than 5% of the sample. Table 2 shows the values of the stringency score and 7-days death incidence for the sampled countries for the period under analysis. The most severe measures were in place in Portugal and Germany, while Luxemburg and Lithuania experienced the least stringent ones. The Supplementary Table SA1 shows the details by restriction type, showing, e.g., that the higher stringency scores in Germany and Portugal were driven by stronger restrictions on gatherings and international travels.

**TABLE 1:** Sample characterization (own calculation using Share data; Europe, 2020).

Variables	N (%)	Depression	Anxiety	Trouble sleeping	Feeling loneliness
Total					
50–64	11,136 (29.15)	15.4	24.0	9.0	9.1
65–79	20,164 (52.78)	15.3	22.2	7.6	11.1
≥80	6,905 (18.07)	19.9	24.1	8.3	16.8
Female	22,491 (58.63)	19.8	27.2	9.5	13.9
Male	15,867 (41.37)	11.1	17.5	6.2	8.1
Non-worker	30,154 (78.96)	17.0	23.4	8.2	12.7
Worker	8,037 (21.04)	13.2	22.0	7.8	7.2
Not living alone	28,833 (75.17)	15.0	22.6	7.9	8.8
Living alone	9,525 (24.83)	19.6	24.5	8.9	19.7
No close death	37,222 (97.63)	15.9	22.8	8.0	11.3
Close death	903 (2.37)	27.8	36.2	14.6	18.1
No close hospitalization	36,895 (96.88)	15.9	22.7	8.0	11.3
Close hospitalization	1,187 (3.12)	24.5	34.7	12.4	16.3
Hip fracture	176 (46.0)	16.1	23.0	8.1	11.5
Diabetes or high blood sugar	753 (1.96)	15.9	22.9	8.0	11.4
High blood pressure or hypertension	1,794 (4.68)	15.6	22.4	7.7	11.1
Cancer	582 (1.52)	16.0	22.9	8.0	11.5
Chronic lung disease	458 (1.19)	16.0	22.9	8.1	11.4
Heart attack or other heart problem	1,062 (2.77)	15.8	22.7	7.9	11.3

**TABLE 2:** Mean stringency index score and 7-days death rate per million, by country (own calculation using Share data; Europe, 2020).

Country	Stringency score		7-days death incidence	
	Mean	SD	Mean	SD
Belgium	51.4	1.8	0.379	0.211
Bulgaria	37.3	3.1	0.425	0.082
Croatia	50.2	7.2	0.079	0.092
Cyprus	50.9	5.4	0.012	0.035
Czech Republic	36.9	2.5	0.070	0.030
Denmark	56.9	1.1	0.078	0.047
Estonia	35.0	7.6	0.005	0.019
Finland	36.2	2.2	0.015	0.010
France	55.0	8.5	0.242	0.088
Germany	61.3	3.6	0.083	0.024
Greece	49.9	6.6	0.024	0.022
Hungary	54.7	0.8	0.087	0.035
Italy	55.2	3.1	0.395	0.241
Lithuania	28.5	4.6	0.036	0.024
Luxemburg	26.1	3.6	0.043	0.079
Poland	47.6	5.2	0.206	0.052
Portugal	71.5	1.1	0.320	0.105
Romania	46.1	5.4	0.496	0.132
Slovakia	38.5	1.4	0.000	0.000
Slovenia	41.0	3.3	0.025	0.052
Sweden	59.3	0.0	1.725	0.445

**FIGURE 1:**
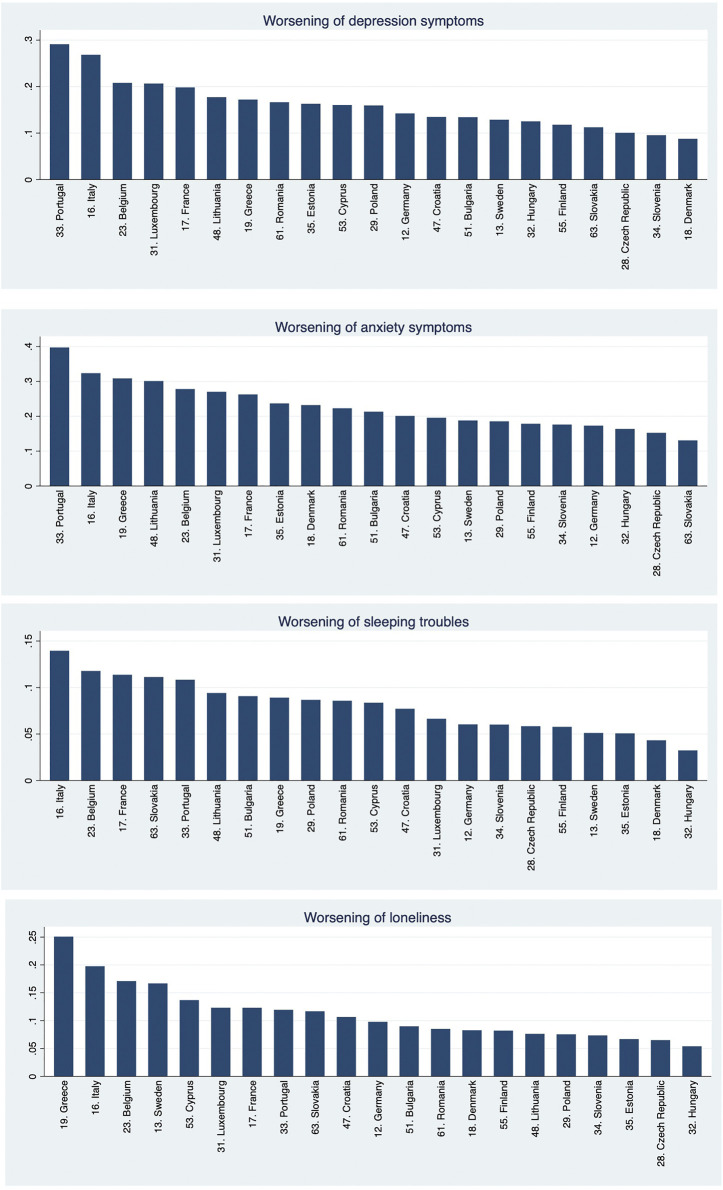
Prevalence of worsening of mental health symptoms, by country (vertical axes represent the percentage of cases) (own calculation using Share data; Europe, 2020).

The worsening of anxiety and depression perception were more common (16.2 and 23.1%, respectively), compared to that of sleeping troubles and loneliness (8.1 and 11.5%, respectively) (Table 3). Note that the anxiety variable was marked by an almost 10% rate of missing responses. The worsening of mental health symptoms’ perception was heterogeneous across countries (Figure 1). Its prevalence seemed higher, at first sight, in Southern Europe countries (Portugal, Italy, Spain, Greece, and Malta).

**TABLE 3:** Frequency of worsening of mental health symptoms (own calculation using Share data; Europe, 2020).

Variables	Frequencies	%	Total (% missing)
Depression	6,157	16.2	38,076 (0.74)
Anxiety	7,998	23.1	34,647 (9.67)
Trouble sleeping	3,107	8.1	38,160 (0.52)
Feeling loneliness	4,379	11.5	38,048 (0.81)

**FIGURE 2:**
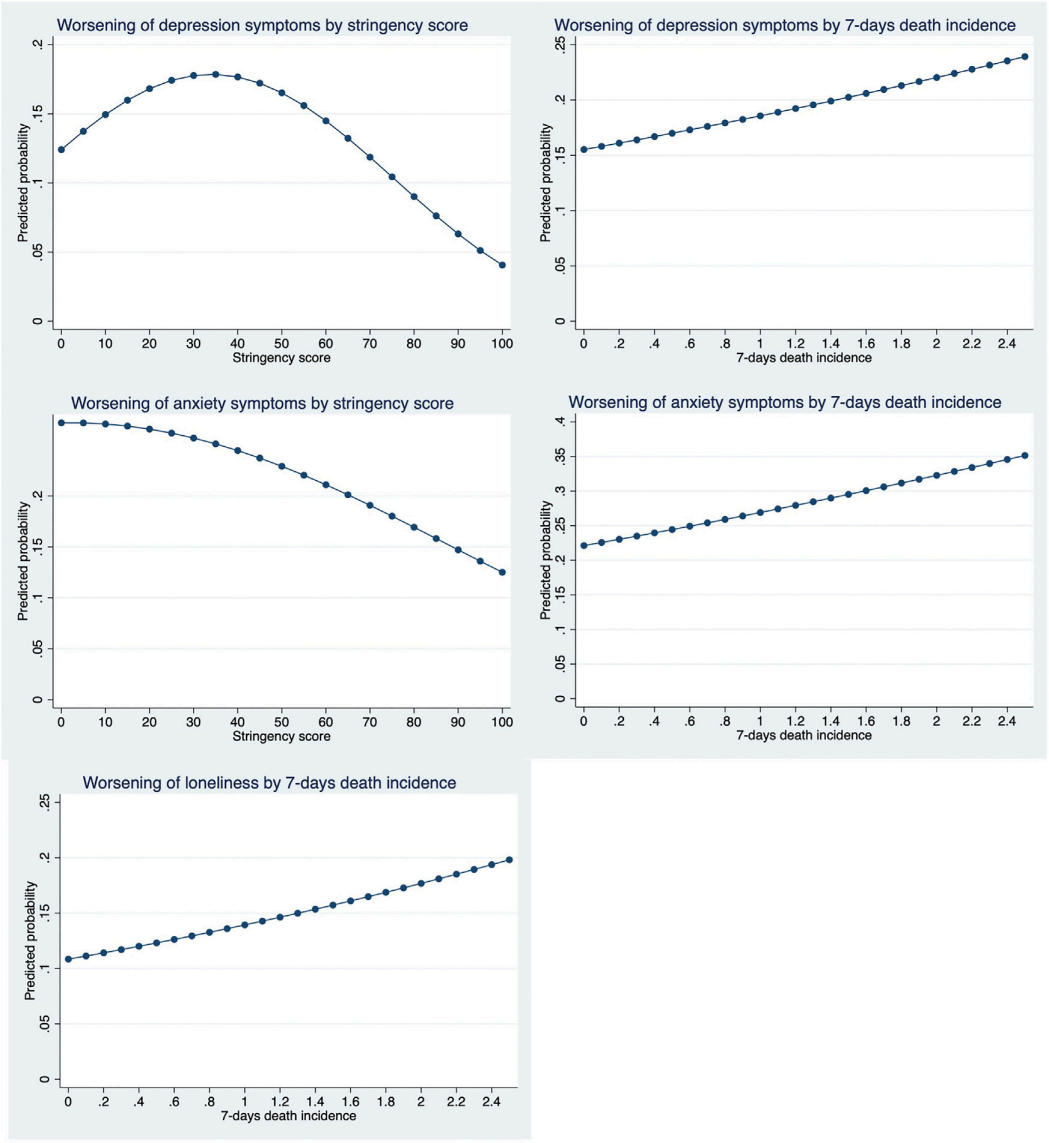
Predicted probabilities, by stringency index score and death incidence (own calculation using Share data; Europe, 2020).

The worsening of depression and anxiety perception was negatively related to the stringency of public health measures, but the relationship was concave in the case of depression (Figure 2; Table 4). No significant association was observed for sleeping troubles or loneliness. The death incidence was positively linked to all symptoms’ perception except sleeping troubles. Figure 2 depicts the magnitude of associations using predicted probabilities. We clearly observe a concave relationship of stringency with depression, from a 16.8% prevalence at a 20-stringency score, a plateau at a 35-stringency score (17.8% probability), followed by a fall to 6.3% probability at 90-stringency score. A ten-point increase in stringency increases the risk of depression worsening perception by 1.6 percentage points in the increasing part of the shape; and decreases it by 2.7 percentage points (pp) in the decreasing part of the shape. Note that only three countries fall below the 40-points stringency score, where the relationship is positive. The 7-days death incidence augmented the risk of depression by 0.29 percentage points per 0.1 additional deaths per million. The risk increased from 15.5 to 23.9% between the lowest and the highest incidence. It raised the risk of loneliness from 10.8 to 19.8%, and that of anxiety from 22.1 to 31.1%. Country-specific marginal effects are available in Supplementary Table SA2).

**TABLE 4:** Marginal effects (ME) of worsening of MH symptoms [standard errors (SE) between brackets]^a^ (own calculation using Share data; Europe, 2020).

	Depression	Anxiety	Trouble sleeping	Loneliness
	ME(SE)	ME (SE)	ME (SE)	ME (SE)
Stringency index score (/100)	−0.140 (0.039)***	−0.162 (0.047)***	−0.026 (0.030)	−0.042 (0.034)
Death incidence per 1 M	0.029 (0.013)**	0.046 (0.016)***	−0.004 (0.009)	0.029 (0.011)***
50–64	References	References	References	References
65–79	−0.011 (0.005)**	−0.022 (0.006)***	−0.014 (0.004)***	−0.003 (0.004)
≥80	0.016 (0.007)**	−0.018 (0.008)**	−0.014 (0.005)***	0.019 (0.006)***
Male	−0.085 (0.004)***	−0.098 (0.004)***	−0.032 (0.003)***	−0.048 (0.003)***
Worker	−0.023 (0.005)***	−0.014 (0.006)**	−0.005 (0.004)	−0.032 (0.005)***
Living alone	0.024 (0.004)***	0.001 (0.005)	0.008 (0.003)**	0.093 (0.005)***
Close death	0.047 (0.014)***	0.060 (0.017)***	0.027 (0.011)**	0.017 (0.011)
Close hospitalization	0.027 (0.012)**	0.055 (0.015)***	0.008 (0.009)	0.014 (0.010)
Hip fracture	0.061 (0.032)**	0.065 (0.038)*	0.051 (0.024)**	0.001 (0.021)
Diabetes or high blood sugar	0.027 (0.014)*	0.017 (0.018)	0.020 (0.011)*	0.007 (0.012)
High blood pressure or hypertension	0.061 (0.012)***	0.087 (0.015)***	0.048 (0.010)***	0.050 (0.010)***
Cancer	0.083 (0.018)***	0.077 (0.021)***	0.031 (0.013)**	−0.029 (0.011)***
Chronic lung disease	0.076 (0.019)***	0.092 (0.024)***	0.012 (0.012)	0.037 (0.016)**
Heart attack or other heart problem	0.074 (0.014)***	0.088 (0.017)***	0.037 (0.011)***	0.028 (0.011)**

a All analyses include country fixed effects (in appendix); ***p < 0.01, **p < 0.05, *p < 0.10.

Other relevant results are worth highlighting for covariates. For depression, the risk was significantly lower among those aged 65–79 compared to younger people, but higher among those aged 80 and above. For loneliness, the risk was significantly higher among those above 80. Yet, for anxiety and sleeping troubles, the risk fell significantly at 65 years old, inclusively above 80.

Also, all risks were significantly lower among men and among people who were workers before the pandemic. On the contrary, all risks were enhanced among people living alone, or who lost a close person due to the pandemic. Depression and anxiety worsening perception were also significantly related to the hospitalization of a close person. The occurrence of a comorbidity in the last 3 years was significantly associated with a worsening of all symptoms’ perception in almost all cases.

Interactions were tested between stringency and death incidence and all variables. To facilitate the analyses and interpretations, morbidities were recoded as dichotomous variables (no morbidity vs. at least one morbidity). Only three of 28 interactions were statistically significant (Supplementary Table SA3). When subject to greater stringency, people living alone were more affected by anxiety symptoms’ perceptions, and people aged 65–79 and with morbidities were less affected by sleeping troubles perceptions.

We also examined the possible cause of the stringency-depression relationship, by testing the effect of the stringency duration, which was negative (Supplementary Table SA4). That is, for a 10-day additional stringency duration, the risk of depression perception growing worse fell by 0.026 pp (a statistically significant effect of low magnitude). When decomposing the stringency score into its various components, we observed a positive significant link with the closing of public transport, and a negative significant link with the cancelation of public events, school closing, and international travel restrictions.

Finally, we tested the influence of stringency and death incidence without including country fixed effects (Supplementary Table SA5). As expected, all stringency scores became statistically significant, with a negative link for depression and anxiety perception, and a positive link for sleeping troubles and loneliness. We cannot consider these effects to be entirely reliable, as they possibly capture unobservable country effects, but as the highest association with stringency in a less conservative approach.

## Discussion

### Key Findings

Our findings show that in a large sample of European mature adults and elderly, the worsening of depression and anxiety symptoms perception during the pandemic affected fewer than one fifth and one fourth of the respondents, respectively. By contrast, the exacerbation of sleeping troubles and feelings of loneliness affected only one in ten persons. The worsening of depression perception was positively related to the stringency of public health measures only when these were weak; for more severe measures, such as those imposed in most European countries, stronger stringency was related to a decreased prevalence of depression worsening. The worsening of anxiety perception was negatively related to the stringency of measures. By contrast, all symptoms’ perception were aggravated by the severity of the pandemic as proxied by the death incidence.

Risks decreased with age for all outcomes but increased after 80 years old for depression and loneliness perception. All risks were lower among men and among people who had been working before the pandemic, but higher among people living alone, or who experienced a close COVID-related death. With few exceptions, relationships with stringency and death incidence did not vary significantly across individual characteristics.

### Interpretation

The results partly confirm those of earlier studies, which did not show a substantial change in mental health conditions during the first months of the pandemic among the elderly [1, 3–6, 11]. Unlike earlier longitudinal or repeated cross-section research, our study did not compare mental health symptoms with the pre-COVID-19 period but was instead based on the self-reporting of symptoms worsening during the last month. On the one hand, this measurement may be less accurate because people may have forgotten their precise psychological condition before the pandemic; on the other hand, our measurement allows identifying with greater precision the change in the emotional status over a very specific period, reducing the confounding influence of other more long-term factors. It also avoids the bias of repeated cross-sections, i.e., the possible change in respondents’ characteristics. Also, by considering a cross-country sample, we could assess more precisely the link between symptoms’ worsening and the country-specific context. By doing so, our ability to relate outcomes to the specificities of the COVID-19 situation is strengthened, thereby potentially reducing the bias of other unrelated changes that may have occurred.

The weak or inverse link with control measures highlights the possible resilience of mature adults and the elderly. Semi-structured qualitative interviews showed that the elderly were more worried about COVID-19 infection than about isolation, that they adopted coping strategies based on prior experience(s) with depression (regular schedules, distractions, mindfulness, continued—virtual—social interaction, access to mental healthcare), and that “they would rather shelter-in-place than risk getting the virus” [12]. The proactive coping was observed to be more common among older adults, including a better knowledge about the virus, which also protected them from COVID-19-related stress [13]. The same study depicted the greater anxiety about becoming infected among older people. These results would explain why more stringent measures reduce mental health symptoms—by improving the sense of protection—while the death incidence boosts them—by raising the specter of infection. Also, these earlier findings may explain the strong link between all symptoms and a close COVID-related death.

None of our other findings are particularly surprising. The literature has long identified that women are more at risk of depression than men [14]; this finding was also observed among older European people using the SHARE data [15]. The gender gap has also been long demonstrated regarding anxiety [16] and insomnia [17]. Social support and social networks have been shown to protect individuals from depression [18]. Although living alone does not imply the absence of social support, it is likely to be related, especially among retired people, explaining the link between living alone and mental health symptoms. By contrast, the link between non-working (mostly retirement in our case) and mental health was unclear in the literature [19]. Finally, chronic diseases are well-known predictors of depression and anxiety among the elderly [20].

A major social concern during lockdowns has been the mental health of the elderlies, who may suffer, more than other groups, from isolation and anxiety due to the risks of the disease. Our study highlights that this concern might have been excessive, as we did not observe a major deterioration of mental health symptoms’ perception. Based on recent studies, it seems that the major priority, from a public mental health perspective, should be instead interventions targeting younger populations. Further research should be devoted to understanding the precise factors that protect the mental health of older populations when facing such stressing events.

### Strength and Limitations

The major strength of our paper is the use of data from a representative sample of European mature elderly persons, from a large array of countries. Another major strength is the possibility of identifying the epidemiologic and public health policy context in place at the moment people were interviewed.

A major limitation is that outcomes were measured in a relatively simplistic way, asking people their perceptions about worsening of the mental health condition. These are surely less reliable indicators than the specific depression or anxiety scales that are commonly used in the literature. Nevertheless, the coincidence of relationships reported in earlier studies provide some confidence about the validity of indicators.

Another limitation is that the SHARE survey took place in June and July, during which the burden of COVID-19 and the public health measures were the weakest in 2020, so that we might not have captured the individual response to more difficult contexts. Still, the heterogeneity across Europe was sufficient to identify significant links of symptoms with the incidence of both stringency and death.

Also, we used the 7-days mortality rate as explanatory variable, but no information was accessible about how this information was displayed in all countries by the media, hence affecting people’s concerns. Note however that newspapers have certainly addressed this issue in the most critical periods of the pandemic, referring to the mortality burden and occupancy rates of hospital beds. Note also that the significant results we obtained certainly highlight that people were aware and affected by the situation.

Finally, the collection of the data used occurred during the first wave of the pandemic. The literature mentions the existence of an “emotionally honeymoon phase of disaster response” in the short term, which may have vanished in the following months of the pandemic, as restrictions and infections remained high [12]. Interviews with elderly people indeed referred to concerns about long-term consequences [12]. Note, however, that our results indicate that the worsening of mental health symptoms’ perception decreased with the duration of restrictions, possibly questioning the severity of the long-term impact.

### Conclusion

Exposure to control measures to combat COVID-19 did not severely affect the mental health of European adults aged 50 and above. By contrast, the worsening of mental health symptoms’ perception was linked to the COVID-19 mortality burden. Older people were the most hit by the pandemic and were more affected psychologically by the COVID-19 epidemiological situation, and control measures were protective (or neutral) to their mental health condition.
