# Supplementary material for: How do European Mature Adults and Elderly Perceive SARS-COV-2 and Associated Control Measures? A Cross-Country Analysis of Mental Health Symptoms in June and July 2020
Source: Int J Public Health. 2022 Feb 23;67:1604218. doi: 10.3389/ijph.2022.1604218 (PMC8906259; doi:10.3389/ijph.2022.1604218)
Supplement: Supplementary file 1 [file DataSheet1.docx]

# Appendix

Table A1. Mean stringency index score by type of restriction, by country (own calculation using Share data; Europe, 2020)

| Country | School closing | Workplace closing | Cancel public events | Restriction on gatherings | Close public transport | Stay-at-home require. | Restriction internal movement | International travel controls |
| --- | --- | --- | --- | --- | --- | --- | --- | --- |
| RANGE | 0-3 | 0-3 | 0-2 | 0-4 | 0-2 | 0-3 | 0-2 | 0-4 |
| Belgium | 1.632 | 2.015 | 2.000 | 3.016 | 0.000 | 0.032 | 0.027 | 3.000 |
| Bulgaria | 2.000 | 1.000 | 1.117 | 1.261 | 0.000 | 0.000 | 0.000 | 2.717 |
| Croatia | 2.000 | 2.000 | 0.657 | 1.669 | 0.835 | 1.000 | 0.000 | 2.760 |
| Cyprus | 2.000 | 0.312 | 1.156 | 2.589 | 1.000 | 1.000 | 0.000 | 3.000 |
| Czech Republic | 1.187 | 1.000 | 1.000 | 1.416 | 0.000 | 0.000 | 0.604 | 3.000 |
| Denmark | 2.000 | 2.000 | 1.000 | 2.807 | 1.000 | 1.000 | 0.000 | 3.000 |
| Estonia | 1.000 | 1.119 | 0.560 | 2.652 | 0.000 | 0.000 | 0.000 | 2.000 |
| Finland | 1.000 | 1.000 | 1.187 | 1.000 | 0.000 | 0.000 | 0.000 | 3.000 |
| France | 1.187 | 1.187 | 1.879 | 4.000 | 0.187 | 0.187 | 1.187 | 3.000 |
| Germany | 2.494 | 2.000 | 2.000 | 3.749 | 0.000 | 0.000 | 2.000 | 3.000 |
| Greece | 1.199 | 0.000 | 0.833 | 3.000 | 1.000 | 1.000 | 1.000 | 2.430 |
| Hungary | 2.000 | 1.016 | 1.016 | 1.984 | 1.000 | 1.000 | 1.000 | 3.000 |
| Italy | 3.000 | 1.963 | 2.000 | 1.793 | 0.000 | 0.963 | 0.000 | 2.189 |
| Lithuania | 1.000 | 0.073 | 0.037 | 2.024 | 1.000 | 0.037 | 0.037 | 2.614 |
| Luxemburg | 1.000 | 0.731 | 0.731 | 0.655 | 0.000 | 0.731 | 0.004 | 0.000 |
| Poland | 2.000 | 2.000 | 0.691 | 2.000 | 0.691 | 0.000 | 0.000 | 3.037 |
| Portugal | 2.000 | 2.000 | 2.000 | 3.842 | 1.000 | 1.730 | 1.730 | 3.000 |
| Romania | 2.000 | 1.408 | 1.408 | 2.000 | 0.000 | 0.120 | 0.000 | 3.090 |
| Slovakia | 2.000 | 0.000 | 0.000 | 2.000 | 0.998 | 0.025 | 1.000 | 2.163 |
| Slovenia | 1.000 | 0.241 | 2.000 | 2.120 | 0.000 | 0.000 | 0.000 | 3.000 |
| Sweden | 2.000 | 1.000 | 2.000 | 3.000 | 1.000 | 1.000 | 0.000 | 3.000 |

Table A2. Marginal effects of country fixed effects (own calculation using Share data; Europe, 2020)

|  | Depression | Anxiety | Trouble | Loneliness |
| --- | --- | --- | --- | --- |
| Belgium | 0.017 | 0.057 | 0.122 | 0.041 |
| Bulgaria | -0.053 | -0.015 | 0.190 | -0.028 |
| Croatia | -0.030 | 0.010 | 0.136 | 0.007 |
| Cyprus | -0.014 | 0.001 | 0.179 | 0.028 |
| Czech Republic | -0.085 | -0.064 | 0.160 | -0.052 |
| Denmark | -0.071 | 0.054 | 0.147 | -0.017 |
| Estonia | -0.019 | 0.026 | 0.156 | -0.048 |
| Finland | -0.062 | -0.033 | 0.182 | -0.022 |
| France | 0.028 | 0.066 | 0.120 | 0.004 |
| Greece | 0.005 | 0.124 | 0.128 | 0.145 |
| Hungary | -0.041 | -0.025 | 0.229 | -0.056 |
| Italy | 0.099 | 0.121 | 0.115 | 0.090 |
| Lithuania | -0.008 | 0.073 | 0.194 | -0.037 |
| Luxembourg | 0.031 | 0.044 | 0.233 | 0.025 |
| Poland | -0.018 | -0.023 | 0.129 | -0.029 |
| Portugal | 0.201 | 0.249 | 0.170 | 0.037 |
| Romania | -0.018 | 0.003 | 0.158 | -0.028 |
| Slovakia | -0.055 | -0.069 | 0.167 | 0.027 |
| Slovenia | -0.081 | -0.028 | 0.148 | -0.036 |
| Sweden | -0.068 | -0.055 | 0.277 | 0.000 |

Table A3. P-values of interactions with stringency and death incidence variables. (own calculation using Share data; Europe, 2020)

|  | Depression | | Anxiety | | Sleeping trouble | | Loneliness | |
| --- | --- | --- | --- | --- | --- | --- | --- | --- |
|  | Stringency | Death | Stringency | Death | Stringency | Death | Stringency | Death |
| 50-64 | . | . |  |  |  |  |  |  |
| 65-79 | 0.215 | 0.483 | 0.368 | 0.313 | **0.094** | 0.405 | 0.829 | 0.720 |
| >=80 | 0.681 | 0.388 | 0.755 | 0.469 | 0.570 | 0.818 | 0.522 | 0.649 |
| Male | 0.498 | 0.617 | **0.084** | 0.467 | 0.264 | 0.683 | 0.701 | 0.118 |
| Worker | 0.970 | 0.826 | 0.265 | 0.567 | 0.227 | 0.956 | 0.925 | 0.632 |
| Alone | 0.162 | 0.974 | **0.011** | 0.226 | 0.921 | 0.534 | 0.833 | 0.268 |
| Close death | 0.800 | 0.445 | 0.720 | 0.798 | 0.522 | 0.749 | 0.335 | 0.524 |
| Any morbidity | 0.267 | 0.645 | 0.871 | 0.758 | **0.016** | 0.168 | 0.399 | 0.841 |

Table A4. Marginal effects for other stringency-related variables, worsening of depression^1^. (own calculation using Share data; Europe, 2020)

|  | ME | (SE) |  | ME | (SE) |  |
| --- | --- | --- | --- | --- | --- | --- |
| Stringency index score (/100) | 0.353 | (0.184) | * | . |  |  |
| Stringency^2 index score (/100) | -0.004 | (0.002) | ** | . |  |  |
| Death incidence per 1M | 0.021 | (0.014) |  | 0.012 | (0.147) |  |
| Stringency duration (days/100) | -0.026 | (0.008) | *** | . |  |  |
| School closing (1-3 score) | . |  |  | -0.010 | (0.006) | * |
| Workplace closing (0-3 score) | . |  |  | 0.007 | (0.006) |  |
| Cancellation of public events (0-2 score) | . |  |  | -0.015 | (0.006) | ** |
| Restrictions in gatherings (0-4 score) | . |  |  | -0.002 | (0.005) |  |
| Closing public transport (0-1 score) | . |  |  | 0.034 | (0.012) | *** |
| Stay-at-home requirements (0-2 score) | . |  |  | -0.019 | (0.013) |  |
| Restrictions in internal movement (0-2 score) | . |  |  | -0.001 | (0.013) |  |
| International travel control (0-4 score) | . |  |  | -0.028 | (0.006) | *** |

Note: ^1^all analyses include the complete set of covariates; *** p<0.01 * p<0.05, * p<0.1

Table A5. Marginal effect (ME) of worsening of MH symptoms (standard errors (SE) between brackets), without country fixed effects.^1^ (own calculation using Share data; Europe, 2020)

|  | Depression |  |  | Anxiety |  |  | Trouble sleeping | |  | Loneliness |  |  |
| --- | --- | --- | --- | --- | --- | --- | --- | --- | --- | --- | --- | --- |
|  | ME | (SE) |  | ME | (SE) |  | ME | (SE) |  | ME | (SE) |  |
| Stringency index score (/100) | -0.400 | (0.120) | *** | -4.539 | (0.827) | *** | 3.283 | (0.099) | *** | 8.421 | (1.188) | *** |
| stringency^2 index score (/100) | 0.005 | (0.001) | *** | -0.053 | (0.009) | *** | -0.002 | (0.001) | *** | -0.007 | (0.001) | *** |
| Death incidence per 1M | 0.024 | (0.005) | *** | 0.090 | (0.038) | ** | 0.009 | (0.004) | ** | 0.027 | (0.004) | *** |

Notes: ^1^all analyses include all covariates but country fixed effects; *** p<0.01 * p<0.05, * p<0.1
